# Understanding sexual violence: Perspectives from an adolescent HIV prevention study

**DOI:** 10.4102/jphia.v16i1.750

**Published:** 2025-09-17

**Authors:** Nadia Ahmed, Emily Webb, Richard Muhumuza, Andrew S. Ssemata, Millicent Atujuna, Lynda Stranix-Chibanda, Teacler Nematadzira, Janan J. Dietrich, Gugulethu Tshabalala, Stefanie Hornschuh, Helen A. Weiss, Janet Seeley, Julie Fox

**Affiliations:** 1 Department of Genito-Urinary Medicine, Central North West London NHS Trust, London, United Kingdom; 2 Faculty of Health Sciences, Desmond Tutu HIV Foundation/Centre, University of Cape Town, Cape Town, South Africa; 3 Department of Infectious Diseases Epidemiology, Faculty of Epidemiology and Population Health, London School of Hygiene and Tropical Medicine, London, United Kingdom; 4 Medical Research Council/Uganda Virus Research Institute and London School of Hygiene and Tropical Medicine Uganda Research Unit, Entebbe, Uganda; 5 Clinical Trials Research Centre, Faculty of Medicine and Health Sciences, University of Zimbabwe, Harare, Zimbabwe; 6 Child and Adolescent Health Unit, Faculty of Medicine and Health Sciences, University of Zimbabwe, Harare, Zimbabwe; 7 Perinatal HIV Research Unit, School of Clinical Medicine, Faculty of Health Sciences, University of the Witwatersrand, Johannesburg, South Africa; 8 Department of Global Health and Development, Faculty of Public Health and Policy, London School of Hygiene and Tropical Medicine, London, United Kingdom; 9 Department of Social Science, Africa Health Research Institute, Durban, South Africa; 10 Department of Infectious Diseases, Faculty of Medicine, Kings College London, London, United Kingdom

**Keywords:** sexual violence, rape, sexual assault, HIV, prevention

## Abstract

**Background:**

Sub-Saharan Africa has a high prevalence of sexual violence in young women, with less data on young men.

**Aim:**

We investigated the prevalence of forced sex among adolescents and young people and described factors putting them at risk of sexual violence.

**Setting:**

The study was conducted in South Africa, Uganda and Zimbabwe.

**Methods:**

We conducted a cross-sectional, structured survey among 1330 13–24-year-old male and female participants. Logistic regression models were used to estimate odds ratios for associations with forced sex, adjusting for site, sex and age. Sixty in-depth interviews and 24 group discussions were also conducted. Data were transcribed, translated and analysed using thematic framework analysis.

**Results:**

Seventy-six out of 1326 participants (6%) reported forced sex in the last 6 months. Forced sex was most commonly reported in Entebbe versus other sites, female than male participants, and 18–24 years than 13–18 years. Associations were seen with younger sexual debut (adjusted odds ratio [aOR]: 0.89; 95% confidence interval [CI]: 0.81, 0.98), ever having transactional sex (aOR: 2.18; 95% CI: 1.19, 4.02), risk-taking (aOR: 3.51; 95% CI: 1.99, 6.19), depression (aOR: 3.20; 95% CI: 1.69, 6.06), anxiety (aOR: 2.07; 95% CI: 1.08, 3.96) and binge drinking (aOR: 2.66; 95% CI: 1.33, 5.36), and strong association with forcing someone to have sex (aOR: 7.54; 95% CI: 3.68, 15.46). Qualitative data support these results.

**Conclusion:**

Our findings identify risks similar to those for sexual violence.

**Contribution:**

We suggest protection strategies to police times and places of risk are developed, and addressed in economic and legal country specific guidance.

## Background

Globally, over 1.4 billion children and adolescents aged 2–17 years experienced physical, emotional and/or sexual violence, according to population survey data.^1^ Data specifically on sexual violence in children, adolescents and young people (AYP) are limited, with only 57 countries reporting estimates on national prevalence for girls (median 3%: range 0–25) and only 11 for boys (median 1%: range 0–4), from 2012 to 2019.^2^ Violence is high in both the Global South and North, but sexual violence disproportionately affects those living in low- and middle-income countries, with sub-Saharan African (SSA) countries being third in the global prevalence of sexual violence in young females (33%), to South East Asia (35%) and Oceania (51%).^3^ While South Africa as a whole has the highest rate of rape in the world, five times higher than in Zimbabwe and 66 times higher than Uganda^4^ data on AYP as survivors of sexual violence, while sparse, show a different picture: 15% of girls and 10% of boys aged 13–17 years in a nationally representative cross-sectional study in South Africa,^5,6^ 33% of females aged 18–24 years and 9% in male counterparts from data from the Zimbabwe National Statistic Agency,^7,8^ and 25% and 35% in females and 11% and 17% in males, aged 13–17 years and 18–24 years, respectively from the Ugandan Violence Against Children Survey.^9^ Sexual violence in AYP remains prevalent, as these data show, with literature on male victims being particularly scarce because of similar reasons for reporting as for females, namely, fear, embarrassment, stigma, not being believed, but more so because of harmful gender stereotype and expectations around masculinity.^10^

A form of gender-based violence, sexual violence is defined by the World Health Organization as:

[*A*]ny sexual act, attempt to obtain a sexual act, unwanted sexual comments or advances, or acts to traffic, or otherwise directed, against a person’s sexuality using coercion, by any person regardless of their relationship to the victim, in any setting, including but not limited to home and work.^11^

Sexual violence of AYP tends to occur together and with other forms of violence, from physical assault to forced exposure to sexual images. Perpetrators have many different underlying motivations, but sexual interests (paraphilia) and cognitive distortions tend to be two common factors. Presentation of sexual violence in minors is complex, ranging from overt symptoms or signs to delayed presentations because of fear of threats made by perpetrators. It is a social and public health concern, but above all, a violation of human rights with devastating physical and psychosocial consequences, impacting the victim and or survivor at an individual, family and community level, with long-lasting effects into adulthood.^12^ However, sexual violence is, as is any other form of violence, preventable, with the responsibility resting not only with governments but society as a whole.

While progress has been made in understanding the extent of sexual violence in AYP, namely, the epidemiology of victims and/or survivors and perpetrators, risk and protective factors, preventative measures, and management, the full scale of this growing problem continues to be under-reported. A recent scoping review^13^ concludes a modest amount of literature on prevalence of mainly sexual violence, with limited interventions and scale-up imperative. On the one hand, this could be attributed to the complexities that surround child and adolescent sexual violence leading to victims and/or survivors not understanding what has happened, fear of reporting for retaliation from the perpetrator, not being believed, lack of specialist services, lack of faith in justice services, as well as stigma and discrimination. On the other hand, the data available typically measure the prevalence of types of violence or focus on single risk or protective factors, while also acknowledging that gathering evidence on this sensitive topic acts as a barrier itself, to documenting the full magnitude of sexual violence against AYP. Primary prevention of sexual violence is particularly sidelined in favour of the implementation of management pathways of victims and/or survivors.

Efforts to prevent sexual violence in AYP in South Africa are limited but more predominant than in other countries within SSA. Interventions in South Africa focused on educational sessions on sexual violence and linked subjects with or without parents or caregivers. One study looked at the impact of cash transfer on school attendance. All bar two out of seven studies showed an impact. Prevention interventions in Uganda, although few, showed a greater variety in strategies; two used mentoring strategies with educational sessions and one involved a creative process to support the learning environment. Zimbabwe had only one study evaluating the impact of cash transfers on youth and their caregivers, inclusive of educational sessions, also showing impact. All the interventions focused on similar strategies, centred around children, adolescents and youth with education and/or mentoring within schools, and economic empowerment.^13^ Gaps are evident on a number of levels ranging from other environments AYP are in (family, schools, community), addressing socio-environmental factors, and interventions regarding laws and policies.

The Combined HIV Adolescent Pre-Exposure Prophylaxis (PrEP) and Prevention Study (CHAPS) was a mixed-methods research study that aimed to investigate the acceptability and feasibility of implementing daily and on-demand PrEP in AYP in SSA, and to determine on-demand dosing schedules for insertive sexual intercourse, to inform the choice of intervention for future Phase III PrEP studies in young people in SSA, and to improve strategies for PrEP implementation. The social science component of CHAPS included qualitative data collection and a quantitative survey. Together, these provide an opportunity to assess, using a mixed-methods approach, the primary objectives of the current analysis: to determine the prevalence of forced sex in male and female AYP and to describe factors putting them at risk of sexual violence in South Africa, Uganda and Zimbabwe.

## Research methods and design

The CHAPS study, as aforementioned, was the overarching study, and the quantitative and qualitative analyses were secondary analyses from the CHAPS study. As such, any reference to the CHAPS study refers to the methods used as the data were analysed from the main CHAPS study.

### Study sites

The social science component of CHAPS was conducted between September 2018 and November 2018 at four research sites: the Perinatal HIV Research Unit (PHRU) in Soweto, Johannesburg; the Desmond Tutu HIV Foundation (DTHF) in Cape Town, South Africa; the Medical Research Council/Uganda Virus Research Institute (MRC/UVRI) and London School of Hygiene and Tropical Medicine (LSHTM) Uganda Research Unit in Wakiso District, Uganda; and the College of Health Sciences Clinical Trials Unit (UZCHS-CTU) at Seke North Clinical Research Site in Chitungwiza, Zimbabwe.

Using a community outreach strategy across all sites, participants were recruited in highly populous informal peri-urban communities, which included informal settlements and areas with low-cost government subsidy housing, also known as Reconstruction and Development Programme (RDP) housing in South Africa and in fishing villages primarily in Uganda. These communities are characterised by high unemployment, low household incomes, overcrowding, limited resources and service delivery.^14^

### Sampling

Trained fieldworkers used a purposive sampling approach to recruit participants from Johannesburg and Cape Town (South Africa), Wakiso (Uganda) and Chitungwiza (Zimbabwe). In Zimbabwe and South Africa, participants were recruited in various community locations such as around schools, taxi ranks, malls, parks, community centres, churches, bars and other public places where young people meet. The CHAPS study fieldworkers provided an overview of the study to establish initial interest. For Uganda, participants were approached in fishing communities in Wakiso District, and study information was provided through local leaders, project mobilisers and village health teams. During the recruitment phase, sampling frameworks were implemented for each country to ensure a pre-specified ratio by age and gender. Data collection was stratified by age (13–17 years and 18–24 years) and gender (male and female) (Table 1).

**TABLE 1:** Sampling framework.

Age group (years)	Number of enrolments		
	*n*	F	M
13–15	58	29	29
16–18	114	57	57
19–24	228	114	114

F, female; M, male.

### Quantitative component

#### Eligibility

Participants were eligible to participate in the survey of the CHAPS study if they were (1) 13–24 years of age, (2) had self-reported sexual intercourse in the past 6 months (South Africa and Zimbabwe only), and (3) were willing to undergo rapid HIV testing to confirm their HIV status. Participants who had a confirmed positive HIV test were provided with support through the study social workers or counsellors and referred to healthcare facilities for ongoing care. Participants who were deemed eligible and tested HIV-negative were enrolled to complete the survey.

#### Data collection procedures

Using Open Data Kit (ODK), a one-off structured online survey was administered by trained interviewers on tablets among 1330 young people across the three countries. The survey was developed collaboratively with South African, Zimbabwean, Ugandan, and UK experts in adolescent health and HIV prevention and treatment. The survey was piloted among adolescent Community Advisory Board members in each country. The survey was available in English and local languages: Zulu and Sesotho in South Africa, Luganda in Uganda, and Shona in Zimbabwe. Interviewers administered the survey in a confidential location identified by the study site and convenient for the participants. The survey lasted approximately 45 minutes. Following any disclosures of forced sex and/or related concerns, study staff did not have access to participants’ responses in the qualitative component but asked participants if there were any experiences or feelings that they would like to discuss; those who said they did want to talk to someone could receive counselling from trained staff at the study clinic or clinic close to the location of the survey.

#### Measures variable

The two outcome variables for the quantitative data analysis were forced sex in the last 6 months and forcing someone to have sex in the last 6 months. To determine forced sex, participants were asked whether in the last 6 months anyone had physically forced them to have sex when they did not want to. For forcing sex, participants were asked whether in the last 6 months they had physically forced someone to have sex with them when that person did not want to. For both outcomes, those responding ‘yes’ were classified as having the outcome, and participants who responded either ‘yes’ or ‘no’ were included in the analysis for that outcome (those who responded with ‘prefer not to answer’ were excluded).

#### Exposure variables

Exposure variables considered for the quantitative analysis were: socio-demographics, including site (Johannesburg, Cape Town, Entebbe, Chitungwiza), sex (male, female), age group (13–15 years, 16–17 years, 18–24 years), and highest level of education (still studying, under Grade 7, Grades 7 to Grade 12 and post-school), money source (employed job, odd jobs, partner, friends, relative, loan, grant), sexual and non-sexual behaviour including age at first sex, whether they had ever had transactional sex, current or recent sexual behaviour including current relationship status (single, boyfriend or girlfriend, married or living as married, separated or divorced, other), age of most recent partner (> 5 years younger, 1–5 years younger, same age, 1–5 years older, > 5 years older), self-perception of risk-taking (takes risks, somewhere in between, avoid taking risks), situations that made the participant think about the risk of acquiring HIV; and mental health measures including depression using the Patient Health Questionnaire (PHQ-9),^14^ anxiety using the Generalized Anxiety Disorder-2 item tool (GAD-2),^15^ symptoms of post-traumatic stress disorder (PTSD) through the Primary Care PTSD Screen for DSM-5 (PC-PTSD-5),^16^ frequency of binge drinking (never, very rarely, less than monthly, monthly, more than weekly) and drug use in the past 30 days.

#### Data analysis

Quantitative data were analysed in Stata version 16 (StataCorp, Texas, United States [US]). The binary outcomes for the analysis was forced sex: yes or no, and forcing someone to have sex: yes or no. Logistic regression was used to calculate crude and adjusted odds ratios (OR) and 95% confidence intervals (CI) for association between each exposure variable and the outcome variables. Adjusted ORs were obtained from multivariable logistic regression models adjusting for age, sex and study setting.

### Qualitative component

#### Eligibility

Twenty-four group discussions (GDs) (eight per country), each consisting of six to eight participants, stratified by gender (male and female) and age (13–17 years and 18–24 years), were conducted between September 2018 and November 2018. Thereafter, 60 in-depth interviews (IDIs) (20 per country) were conducted among participants but not those selected for GDs between November 2018 and February 2019. In this study, participants offered their perspectives on anticipated sexual behaviour changes as a result of introducing PrEP in their community.

#### Data collection and analysis

**FIGURE 1:**
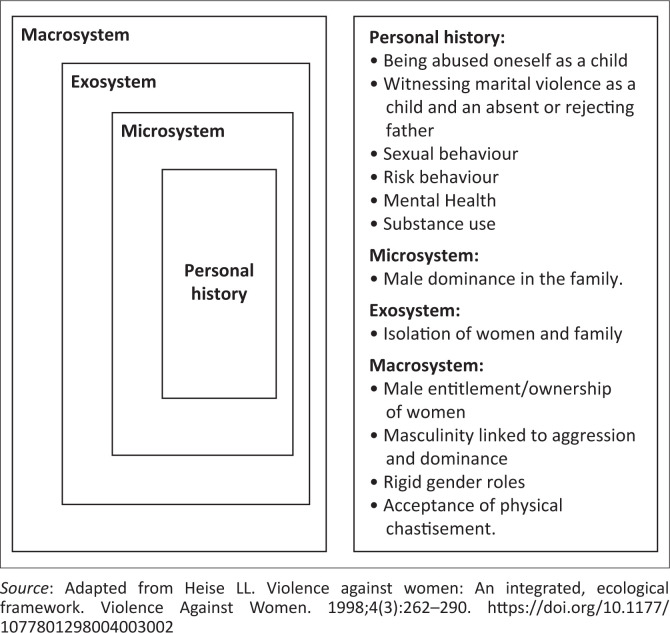
Belsky’s framework.

After providing written informed consent (and assent, where applicable), participants took part in the GDs and IDIs conducted by experienced qualitative researchers, lasting approximately 60 minutes to 120 minutes. The GDs and IDIs were conducted in a conducive and neutral environment that was safe for both the participants and researcher (either in the community or at the research site). A semi-structured guide was used to gather information from participants for both the GDs and IDIs. All GDs and IDIs were audio-recorded, transcribed verbatim, and those conducted in another language were translated into English. To ensure accuracy, the transcripts were cross-checked by the researchers at each site against the audio recordings. Transcripts were coded and analysed manually, drawing on anticipated as well as emergent themes.^17^ The analysis was an iterative process of discussion and revision between co-authors and the research teams. The researchers in each country generated a list of recurrent codes by independently reviewing four transcripts several times and making notes of key ideas and codes. After completing the initial round of coding, the researchers from the three countries discussed the new codes, and these were compared to ensure consistency. Discrepancies in the coding were re-examined, and an initial coding framework and codebook used by all countries were developed after reaching consensus on the final codes. The remaining transcripts were then coded using the codebook. Data were organised by collating emergent themes and to identify recurring patterns and categories in context to the behaviour change research question, using Belsky’s framework,^18^ (see Figure 1) on the aetiology of childhood abuse and neglect to further structure themes. Following any disclosures of forced sex and/or related concerns, study staff asked participants if there were any experiences or feelings that they would like to discuss; those who said they did want to talk to someone could receive counselling from trained staff at the study clinic or a clinic close to the location of the survey.

### Data triangulation

After the analysis of the quantitative survey data in relation to forced sex, the IDI data were reviewed for information on forced sex. These data were compared to the quantitative findings looking for similarities and differences. The qualitative data were then coded using codes drawn from the survey findings and new themes which had only come from the qualitative data.

### Data management

To ensure data confidentiality, participant identifiers were not recorded on study forms and consequently not in the study database. REDCap was used and housed on secured servers and administratively managed by the University of Witwatersrand in Johannesburg, South Africa. User access and database permissions are controlled through unique individual login credentials that are required to be updated every 90 days. The database had an inbuilt audit trail as well, and study data were only shared with the trial statistician. Study data were password-protected and encrypted before transfer.

### Ethical considerations

Ethical clearance to conduct this study was obtained from the University of Cape Town Faculty of Health Sciences Human Research Ethics Committee (No. 290/2018), University of the Witwatersrand Human Research Ethics Committee (No. 180108), London School of Hygiene and Tropical Medicine Research Ethics Committee (No. 17403), Medical Research Council of Zimbabwe (MRCZ/A/2356).

Study procedures were approved as per country requirements: South Africa: University of Cape Town Human Research Ethics Committee, and University of the Witwatersrand Human Research Ethics Committee; Uganda: The Uganda Virus Research Institute Research and Ethics Committee and Uganda National Council for Science and Technology; Zimbabwe: The Joint Research Ethics Committee for the University of Zimbabwe, College of Health Sciences and the Parirenyatwa Group of Hospitals, the Medical Research Council of Zimbabwe and the Research Council of Zimbabwe. Prior to study participation, written informed consent was obtained from participants 18 years and older. Parental consent and participant assent were also required for participants 18 years and younger. Sites in Uganda, Zimbabwe and Cape Town, South Africa, had parental consent waivers in place according to their respective guidelines. Participants were reimbursed for their time and participation based on site national requirements and guidelines.

## Results

### Participant demographics

A total of 1330 participants were included in the quantitative survey. One thousand and twenty six (99.6%) responded to the question of whether they had been forced to have sex in the last 6 months (4 preferred not to answer). A total of 76 out of 1326 (6%) participants reported being forced to have sex in the last 6 months: eight out of 198 (4%) in Johannesburg, 16 out of 400 (4%) in Zimbabwe, 11 out of 237 (5%) in Cape Town, and 41 out of 491 (8%) in Entebbe. In adjusted analysis, forced sex differed by study setting (*p* = 0.02), and was more often reported by female participants (*n* = 45, but also reported by 31 male participants, *p* = 0.07), and more commonly reported with increasing age (*p* = 0.009).

After adjusting for study setting, sex and age group, there was no association with forced sex for the highest level of education, source of income (employed, odd jobs, partner, friends, relative, loan or grant) and household factors (whether the participant was the household head, the age of the household head, number of adults in the household, number of rooms in the household) (Table 2). Group discussions, however, showed other examples of educational and household contexts leading to risks of forced sex. Using Heise’s adoption of Belsky’s framework the following themes were seen:

1. Personal history: Being abused oneself as a child; Exosystem: Isolation of women and family; Macrosystem: Male entitlement/ownership of women, rigid gender roles.

Non-family male members would forcefully engage young girls in sex. This was both at school by the teachers and through travel to and from school:

‘When some girls reach school, they start seducing male teachers. They pursue each other, they may start by comments, “you are smart today you have even put on good shoes, you even ironed today”. The girl will not know that she is seducing the teacher, the next day the teacher will call her and they have sex. After having sex the teacher will tell them not to tell anyone.’ (13–14 years, female, Uganda)

‘I can have sex with guys so that I can get money because I will need money. Even at school if a teacher says, “child I want to have sex with you”, I will not refuse but I will not be having penetrative sex.’ (13–15 years, female, Zimbabwe)

2. Personal history: Being abused oneself as a child; Microsystem: Male dominance in the family.

Participants revealed that some relatives, especially those trusted by the parents of the children to provide childcare during their school holidays, engage girls in forced sex. These children are abused and fear reporting the relatives for fear of causing conflicts in the homes:

‘Another thing, you can go for holidays or they take you to spend your holidays with your relatives. For instance, they can take you to stay at your uncle’s. Personally I fear them because we watch from television and I see that a man who is even your grandfather can forcefully have sex with you.’ (17 years, female, Uganda)

3. Personal history: Witnessing marital violence as a child and an absent or rejecting father.

In families where there is domestic violence, some children run away from home in search of a better place to live without violence. However, in their pursuit of happiness, these children fall into the wrong hands. They may be raped, abused and seem helpless because the conditions might have been even worse than where they have fled from, that is, their own homes:

‘These children of 13 years of age sometimes face violence from parents at home and they run away from home. When they run away from home, they meet different men who end up infecting them after deciding to go out with them or as she runs for refuge she is gang raped.’ (19–24 years, female, Uganda)

**TABLE 2:** Associations between socio-demographic characteristics and forced sex in the last 6 months.

Characteristic	Category	No forced sex, last 6 months		Forced sex, last 6 months		Overall	Adjusted for site, sex and age group		
		*n*	%	*n*	%		Odds ratio	95% CI	*p*
Site	Cape Town	226	95.4	11	4.6	237	0.56	0.28, 1.11	0.020
	Johannesburg	190	96.0	8	4.0	198	0.45	0.21, 0.99	-
	Entebbe	450	91.7	41	8.4	491	Baseline	-	-
	Zimbabwe	384	96.0	16	4.0	400	0.46	0.25, 0.84	-
Sex	Male	641	95.4	31	4.6	672	Baseline	-	0.070
	Female	609	93.1	45	6.9	654	1.55	0.97, 2.49	-
Age group (years)	13–15	144	97.3	4	2.7	148	0.37	0.13, 1.04	0.009
	16–17	232	97.1	7	2.9	239	0.41	0.19, 0.91	-
	18–24	874	93.1	65	6.9	939	Baseline	-	-
Participant is household head	No	1103	94.9	59	5.1	1162	Baseline	-	0.120
	Yes	147	98.6	17	10.4	164	1.67	0.88–3.17	-
Age of household head	Per unit increase	-	-	-	-	-	1.01	0.99, 1.03	0.250
Number of adults in household	1–2	372	93.7	25	6.3	397	Baseline	-	0.450
	3–4	588	94.8	32	5.2	620	1.26	0.70, 2.26	0.200 (trend)
	5+	289	93.8	19	6.2	308	1.53	0.79, 2.98	-
Number of rooms in household	1–2	424	93.4	30	6.6	454	Baseline	-	0.660
	3–4	399	95.0	21	5.0	420	1.05	0.58, 1.92	0.390 (trend)
	5+	427	94.5	25	5.5	452	1.31	0.71, 2.43	-
Number of adults per room in household	< 1	510	95.0	27	5.0	537	Baseline	-	0.610
	≥ 1 and < 2	497	93.8	33	6.2	530	1.11	0.65, 1.92	-
	≥ 2	242	93.8	16	6.2	258	0.81	0.41, 1.61	-
Highest level of education attended	Still studying	663	95.7	30	4.3	693	Baseline	-	0.770
	≤ Grade 7	114	89.1	14	10.9	128	1.50	0.71, 3.14	-
	Grade 7–12	429	93.7	29	6.3	458	1.10	0.62, 1.95	-
	Post-school	44	93.6	3	6.4	47	1.15	0.33, 4.03	-
Money source: employed job	No	1087	94.4	65	5.6	1152	Baseline	-	0.700
	Yes	163	93.7	11	6.3	174	0.88	0.45, 1.72	-
Money source: odd jobs	No	751	93.9	49	6.1	800	Baseline	-	0.520
	Yes	499	94.9	27	5.1	526	0.85	0.51, 1.41	-
Money source: partner	No	977	94.5	57	5.5	1034	Baseline	-	0.870
	Yes	273	93.5	19	6.5	292	0.95	0.52, 1.73	-
Money source: friends	No	1095	94.4	65	5.6	1160	Baseline	-	0.230
	Yes	155	93.4	11	6.6	166	1.55	0.78, 3.08	-
Money source: relative	No	445	92.3	37	7.7	482	Baseline	-	0.580
	Yes	805	95.4	39	4.6	844	0.86	0.51, 1.45	-
Money source: loan	No	1225	94.4	73	5.6	1298	Baseline	-	0.280
	Yes	25	89.3	3	10.7	28	2.16	0.60, 7.74	-
Money source: grant	No	1150	94.2	71	5.8	1221	Baseline	-	0.470
	Yes	100	95.2	5	4.8	105	1.50	0.51, 4.35	-

CI, confidence interval.

### Sexual behaviour

After controlling for age, sex and study setting, behaviour associated with reporting forced sex included younger sexual debut (adjusted odds ratio [OR]: 0.89, 95% CI: 0.81–0.98), ever having transactional sex (adjusted OR: 2.18, 95% CI: 1.19–4.02), and self-perception as a person who takes risks (adjusted OR: 3.51, 95% CI: 1.99–6.19). The relationship status and age of the most recent partner did not show an association with forced sex. There was a very strong association between reporting being forced to have sex and forcing someone to have sex (adjusted OR: 7.54, 95% CI: 3.68–15.46) (Table 3).

**TABLE 3:** Associations between sexual and other behavioural characteristics and forced sex in the last 6 months.

Characteristic	Category	No forced sex, last 6 months		Forced sex, last 6 months		Overall	Adjusted for site, sex and age group		
		*n*	%	*n*	%		Odds ratio	95% CI	*p*
Sexual orientation	Heterosexual	1204	94.2	74	5.8	1278	Baseline	-	0.950
	Homosexual	22	95.7	1	4.4	23	0.73	0.10, 5.60	-
	Bisexual	21	95.5	1	4.6	22	0.89	0.11, 7.05	-
Age at first sex (years)†	-	-	-	-	-	-	0.89	0.81, 0.98	0.020
Last time had sex, how far in advance knew	> 24 h	248	92.9	19	7.1	267	Baseline	-	0.570
	13–24 h	81	90.0	9	10.0	90	1.79	0.76, 4.20	(trend 0.850)
	2–12 h	192	92.3	16	7.7	208	1.37	0.67, 2.79	-
	< 2 h	449	93.4	32	6.7	481	1.13	0.61, 2.08	-
Generally when have sex, how far in advance know	> 24 h	292	93.0	22	7.0	314	Baseline	-	0.600
	13–24 h	98	91.6	9	8.4	107	1.34	0.59, 3.05	(trend 0.340)
	2–12 h	211	94.2	13	5.8	224	0.98	0.48, 2.02	-
	< 2 h	369	92.0	32	8.0	401	1.40	0.78, 2.50	-
Current relationship status	Single	160	93.6	11	6.4	171	0.82	0.41, 1.63	0.210
	Boyfriend/girlfriend	698	92.5	57	7.6	755	Baseline	-	-
	Married/living as married	105	92.9	8	7.1	113	0.51	0.23, 1.14	-
Age, most recent partner	> 5 years younger	13	92.9	1	7.1	14	1.57	0.18, 13.61	0.870
	1–5 years younger	292	93.9	19	6.1	311	1.03	0.45, 2.35	(trend 0.420)
	Same age	199	95.2	10	4.8	209	Baseline	-	-
	1–5 years older	320	90.9	32	9.1	352	1.42	0.63, 3.22	-
	> 5 years older	124	89.9	14	10.1	138	1.50	0.57, 3.93	-
Transactional sex with most recent partner (ever)	No	816	93.4	58	6.6	874	Baseline	-	0.780
	Yes	148	90.2	16	9.8	164	1.09	0.59, 2.00	-
Transactional sex, ever	No	855	93.5	59	6.5	914	Baseline	-	0.020
	Yes	111	87.4	16	12.6	127	2.18	1.19, 4.02	-
Forced someone else to have sex, last 6 months	No	1212	95.1	63	4.9	1275	Baseline	-	< 0.001
	Yes	38	74.5	13	25.5	51	7.54	3.68, 15.46	-
Worried about being forced to have sex, last 3 months	No	1208	96.0	50	4.0	1258	Baseline	-	< 0.001
	Yes	42	61.8	26	38.2	68	16.70	9.15, 30.49	-
Self-perceived risk-taking	Avoid taking risks	715	95.6	33	4.4	748	Baseline	-	< 0.001
	Somewhere in between	302	95.3	15	4.7	317	1.51	0.76, 2.97	-
	Take risks	233	89.3	28	10.7	261	3.51	1.99, 6.19	-
Situation made think might get HIV (past 3 months): alcohol	No	1066	94.6	61	5.4	1127	Baseline	-	0.040
	Yes	184	92.5	15	7.5	199	2.05	1.06, 3.97	-
Situation made think might get HIV (past 3 months): drugs	No	1215	94.5	71	5.5	1286	Baseline	-	0.020
	Yes	35	87.5	5	12.5	40	4.05	1.45, 11.29	-
Situation made think might get HIV (past 3 months): forced sex	No	1210	95.1	63	5.0	1273	Baseline	-	< 0.001
	Yes	40	75.5	13	24.5	53	10.36	4.81, 22.29	-
Depression	No	1044	95.0	55	5.0	1099	Baseline	-	< 0.001
	Yes	206	90.8	21	9.3	227	3.20	1.69, 6.06	-
Anxiety	No	1058	94.6	60	5.4	1118	Baseline	-	0.030
	Yes	192	92.3	16	7.7	208	2.07	1.08, 3.96	-
Frequency, binge drinking	Don’t drink	672	95.7	30	4.3	702	Baseline	-	0.005
	Drink, don’t binge	171	91.0	17	9.0	188	2.48	1.31, 4.71	(trend 0.003)
	< Monthly	167	93.3	12	6.7	179	2.83	1.30, 6.17	-
	At least monthly	236	93.3	17	6.7	253	2.66	1.33, 5.36	-
Drug use past 30 days	No	1094	94.5	64	5.5	1158	Baseline	-	0.060
	Yes	156	92.9	12	7.1	168	1.98	0.96, 4.08	-

CI, confidence interval.

† , Per year increase.

Further review of the qualitative data revealed possible reasons for early sexual debut and transactional sex. Participants revealed having known or learnt about sex at a very young age, and this was because of what they experienced as they grew up. For example, some grow up staying with their parents in a single room and observing everything that happens at night, which the children get to hear or learn from. These living conditions may expose young people to early sexual experimentation:

‘Some parents stay in single roomed houses which they divide with a curtain and the child grows up seeing what the parents always do. So by the time that child grows up, he has taught everyone in the community of their ages. By the time they approach the age of 12, 14 years, they no longer fear to approach even older women for sex.’ (19–24 years, female, Uganda)

Poverty may also encourage some of the AYP to do some things that they would not have done, for example, commercial sex work or sex for money. This was seen for girls and older men, as well as boys and older women, who see their peers earning from it and want to copy:

‘Adolescents in this community love money and jobs are scarce. There are older rich women in this area who opt to engage in sexual activity with young boys. Adolescents here use illicit drugs and they rape girls. When you go to the disco hall and he asks you for sex without a condom, he has to pay 100 000shs and if they don’t have such money then the boy comes and rapes you.’ (17 years, female, Uganda)

‘I think this is not only about girls going out with blessers [*older male partners*] only it also involves boys they are some going out with older women. I don’t know what they call them they will be out with them and also getting money from them. So that boy will be my boyfriend I don’t know whether they use protection or what so it will be good even if I am not going out with blessers to be taking Prep pills.’ (22–24 years, female, Zimbabwe)

### Risk perception

Participants who had a propensity to take risks were more likely to talk about forced sex. Situations that made participants think they might acquire HIV, namely alcohol use, drug use, as well as forced sex itself, were all associated with reporting forced sex. HIV was specifically mentioned in relation to the risk of acquiring HIV as a result of rape:

‘Some youths take alcohol and get drunk, so after getting drunk, they go to the lodge not knowing that they can contract HIV from there … I could go get a boyfriend without knowing his HIV status, I ask him for results but he declines and forces me to sleep with him, and I easily get infected.’ (19–24 years, female, Uganda)

‘I think that we should take them [*PrEP*] for the whole life because these days it is easy to be raped. You can be raped without having taken PrEP saying that I don’t engage in sexual behaviour. But you can be raped by a person who has HIV or AIDS. If that pill is available, it can protect you, so we should take it for the whole life.’ (13–15 years, female, Zimbabwe)

Further comments from participants mentioned PrEP in relation to being forced to have unprotected sexual intercourse, while another confused PrEP with post-exposure prophylaxis in relation to rape. While it is known that alcohol and drug use can impair judgement, adding to risk-taking and its consequences, it is concerning that the risk of rape is considered as a rationale for PrEP.

### Mental health

Forced sex was strongly associated with depression but less so with anxiety (adjusted OR: 3.20, 95% CI: 1.69–6.06 and OR: 2.07, 95% CI: 1.08–3.96, respectively).

### Substance use

Alcohol use and drug use were both associated with forced sex, as participants explained during the interviews:

‘For instance, there are those idle adolescents, who take marijuana, cigarettes and drink liquor and it alternates their brain function or makes them abnormal. One day his friends will tell him that that girl is beautiful, so he will get to woo her and she declines. Then he will say “I will now just rape her”, he will base on that even if he rapes you, you will not get infected.’ (17 years, female, Uganda)

‘You are drunk you are vulnerable and can be forced into sex and when you are drunk your reasoning capacity decreases.’ (22–24 years, male, Zimbabwe)

Alcohol use was specifically referred to in relation to consent with different ways of referring to it from mentioning the brain itself, to capacity decreasing, to ‘not knowing what they will be doing’. Being viewed as ‘reckless’ was also mentioned. Additionally, there was reference to different rationales for drinking, mainly around ‘enjoying drinking alcohol’, but also a way to have sex, ‘after getting drunk … it’s a good thing to go and have sex with boys’. Understanding of the impact on consent and consequences included conscious specific ‘maybe someone can get pregnant’ and non-specific concerns ‘what comes next is dealt with later’; younger substance use debut with ‘people these days from 13 years going up … much into intoxicating substances’, and gender risks albeit not clarifying the specific concerns further with ‘boys at risk because boys these days when they drink and got drunk they are not able to control themselves’.

### Masculinity linked to aggression and dominance, and acceptance of physical chastisement

Participants described encounters in relation to sexual assault including exposure of a body part, undressing, squatting and derogatory comments:

‘The man had pulled out his penis and told my sister that “young girl stand there, there’s something I want to tell you”. My sister was new in that landing site. My sister ran to my mother and told her that “mother look at that man is showing me his penis”. Mother asked us to go and confirm if we know the person, when we reached near the man, we recognized that we knew him. When we approached him he told us that “young girls stand there, there’s something I want to tell you”. We ran. When he reached to our mother he lied to her that “I was calling those children to ask them whether that boat is going to the main land because his friend was so sick. When his friend came to see my mother, she asked him whether he was sick. He replied that “whoever told you that was lying to you I have never been sick. That man raped an old woman and is in jail right now.”’ (13–14 years, female, Uganda)

Other young women recounted stories of sexual harassment from drunk men, or verbal abuse from men they had refused to have sex with, or from former boyfriends who taunt them in public.

### Isolation of women and family

Risk of rape at night was noted, with a few comments about the risk at any time. The risks of being at night could be attributed to activities being hidden by the dark, and most people already being intoxicated from the day’s alcohol. Therefore, if a young girl is on her way to collect something for her parents, she can be taken advantage of:

‘If they see you going home, they start following you and if you fail to fight for yourself by running away, they may undress you … They have sex with you … They rape you. At around 15:00 if you don’t ran, they can rape you.’ (14–17 years, female, Uganda)

‘Others get raped, neh, you see … because of they move around at night … And you find that they get raped by a person that they don’t know, they don’t know that he has HIV, you see, others have this thing of you see, raping them and then they get HIV, you see.’ (13 years, female, Soweto)

‘Ahh as a girl I cannot say I am not at risk, I don’t know what I will encounter as I move around I can get raped depending on the time that I move around at the same time at the house where I reside I am safe I do not expect that I can get HIV unless if my partner does something and then forces me to have unprotected sex, that way I could become at risk.’ (21 years, female, Zimbabwe)

While night time was specifically mentioned most frequently, a real sense of rape happening at any time was mentioned along with the mere risk alone acting as a rationale for PrEP:

‘I think [*PrEP should be taken*] every day because you might not know what to expect, like today, I would take it too, you never know when bad luck can strike like rape.’ (20 years, female, Uganda)

Going on short or long journeys was also mentioned as a risk of rape as well as consequences of HIV acquisition, and therefore the need for taking PrEP for this reason:

‘Yes I may want to take PrEP because I can have a journey, if I have a journey I can be raped.’ (21 years, female, South Africa)

Parents were aware of the risks of rape during short journeys, with one participant mentioning their mother stating ‘I pray he may not rape me!’ after sending her daughter to buy fish.

### Male entitlement/ownership of women, masculinity linked to aggression and dominance, acceptance of physical chastisement

There were a variety of comments about appearance as a risk of rape. Participants revealed that to some of the people in the community, age did not matter, but appearance did. So, if a 10-year-old girl was healthy and had already developed breasts, she would be a potential for rape or sexual activity by men. This coupled with perceived indecent dressing in some communities made some people think that the little girls are ready for sex and thus exacerbating the risk for rape:

‘I think 12 years. First of all most girls grow very quickly. You may think that they are 10 years yet they are 13 years, the breasts have grown so fast and when a man looks at her he will develop desire. Some parents show too much love to their children. They buy for kids skimpy clothes, and that’s a young child who is growing rapidly, and is dressing indecently. So tell me why won’t a man admire her and rape her? Because she is young he will deceive her with some little money, the girl is not in a decisive age. So me I think it’s from 12 years and above.’ (19–24 years, female, Uganda)

‘The ages of 15 because most of the times if guys see you wearing revealing clothes and having budding breasts, they will see you as if you are ripe.’ (13–15 years, female, Zimbabwe)

## Discussion

Our study provides an understanding of sexual violence in male and female AYP in South Africa, Uganda, and Zimbabwe. Six per cent of participants overall reported being forced to have sex in the last 6 months, with reports more common in female participants, with increasing age, and in participants from Uganda. Strong associations were seen between reporting being forced to have sex and forcing someone to have sex, as well as with depression and anxiety. Younger sexual debut, ever having transactional sex or more risk-taking, alcohol and drug use were also associated with being forced to have sex. While some of these findings are not unexpected, reports across the literature about sexual violence in AYP vary widely with a paucity in the data available as a whole with many reports up to 10 years old. However, more female compared to male survivors across the age spectrum are consistently well described, with female survivors also the predominant focus concerning advocacy, awareness and education. Our findings increase the body of evidence and offer recent evidence on the current understanding of sexual violence in AYP, but also provide unique insights into the lives of AYP where awareness, risk and management of rape and sexual assault in AYP in sub-Saharan Africa still clearly need strategic attention.

Female participants reported being forced to have sex more than their male counterparts. The focus across the spectrum of understanding and managing sexual violence, regardless of age, has always had a female preponderance. While our data showed female participants reporting rape more commonly, there was a fairly small difference compared to male participants, 6.9% versus 4.6%, respectively. With little data available about male survivors in sub-Saharan Africa and indeed globally, a study from Uganda showed females more likely to report not being willing at sexual debut than males but males more likely to report being survivors of forced sex,^9,19^ while data from South Africa and Zimbabwe show females more likely to report surviving forced sex.^5,6,20,21^ With the greater focus on violence against females, particularly in prevention and management programmes, not to mention the mainstream media, concern with masculine identity, norms and expectations in society, negative reactions by friends, family and healthcare professionals and males less likely to seek medical and psychosocial support, the gender disparity in reporting forced sex is not completely unexpected. Male AYP as victims of forced sex should not be excluded from the dialogue about sexual violence. A recent scoping review on male victims by Langdridge et al.,^22^ showed a breadth of gaps spanning the spectrum needed to fully understand, prevent and manage male victims of sexual violence. Firstly, evidence on victims is limited to distinct demographics and contexts, and secondly, while studies on support are significantly limited, it suggests major challenges to seeking support. As such, even with the evidence that is available, it is insufficient to inform appropriate interventions for male victims. There is an urgent need to research and implement interventions to support male victims, as well as prevent, which would need a multipronged approach at the local, community, national and international levels.

Reporting forced sex increasing with age in AYP is not surprising because of a multitude of reasons, including not understanding what is happening, threats from perpetrators, being ashamed or embarrassed, fear of not being believed, not knowing who to turn to and being afraid of consequences. Higher reports of forced sex in Uganda, with more qualitative comments too, compared to South Africa and Zimbabwe, were unexpected given that South Africa as a whole has the highest rate of rape in the world,^4^ but comparable to higher reports from studies in AYP in Uganda, followed by Zimbabwe and then South Africa. The high overall rate in South Africa may ironically explain this finding from both the qualitative and quantitative components, as forced sex may be so embedded in South Africa that it becomes normalised and justified, so-called ‘rape culture’, and less of an issue to mention and discuss. This notion is clearly a dangerous status quo that we cannot be complacent with, especially in a country that has a growing population of AYP against a background of social and health inequalities. Our data show a possible normalisation of sexual violence now seen in AYP as opposed to adult population; urgent action is needed to turn the tide for this vulnerable group as the parents of the next generations before it is too late.

Risk factors identified in the quantitative data, younger sexual debut, ever having transactional sex, more risk-taking, alcohol and drug use, depression and anxiety, were similar to those known for AYP, as well as across the age spectrum.^5,6,7,8,9^ Sugar mummies, as opposed to sugar daddies, were mentioned more frequently in the qualitative data, adding to three studies on their practices in SSA.^23^ The strong association seen between reporting being forced to have sex and forcing someone to have sex during the adolescent years is not clearly mentioned in the literature on sexual violence in AYP, but the victim and/or survivor-to-perpetrator cycle in abuse is well described, albeit with the link of childhood survivors to adulthood perpetrators,^24,25,26,27^ as opposed to AYP survivors also being perpetrators, as we found. This link warrants further research.

No association with relationship status was a surprising finding, going against the evidence-rich and well-publicised notion that the majority of survivors know their perpetrator.^27^ Financial and household factors did not show a relation to being forced to have sex, also contrary to that seen in other studies,^27^ including boys in poverty being more likely to be survivors and higher risks in AYP in rural dwellings and without a flush toilet in South Africa,^6^ and poverty generally seen as risks to AYP in Uganda^9^ and Zimbabwe.

Alcohol and drug use can be considered synonymous in the dialogue of consent, the core of the definition of rape and sexual assault. A clear association was seen in the quantitative data, with qualitative comments in abundance providing useful insights from AYPs’ actual understanding of consent, how alcohol and drugs impact judgement and behaviour particularly of perpetrators, condom use and the link to consequences of rape such as HIV. Collectively these indicate a lack of a full understanding of basic yet key concepts about sexual violence in AYP, judgemental and stigmatising attitudes about rape survivors among AYP themselves that span the notion of ‘victim-blaming’, but provide useful insights about sexual violence in AYP in SSA. Firstly, not fully understanding what sexual violence is, indicates that the full scale of the problem is likely to be under-reported, consequentially potentially impacting allocation of resources for effective strategies. Secondly, it implies that current strategies may be inadequate in preventing or managing sexual violence in AYP. A systematic review on primary prevention of sexual violence in adolescents found 202 articles, of which none of the 10 included in the analysis were of interventions in SSA.^28^ While females and males were included within the interventions, gender disparities were evident with those in boys focusing on them as perpetrators only, such as equality and gender equality dialogues, positive attitudes and role-play, and reducing high-risk behaviours. We need to be addressing the whole spectrum of sexual violence in AYP, and consider whether the strategies currently used are being reached and understood by AYP, and also their surrounding society where deep, systematic dysfunctions of cultures and social norms have not prevented and do not prevent sexual violence in AYP. If our data are still showing similar themes as well as different and new themes years after national strategies have been recommended and implemented, have these been implemented effectively enough?

Limitations to our study are present. The CHAPS study was a mixed-methods research study to investigate the acceptability and feasibility of implementing daily and on-demand PrEP in adolescents in SSA. Our focus was not specifically on sexual violence, and the questions on this topic were therefore limited. The quantitative data were collected through non-probability-based sampling and thus may not be representative of all young people in these settings.

## Conclusion

Sexual violence remains a serious public health and human rights problem, with significant short- and long-term physical and psychosocial sequelae. The full scale of rape and sexual assault, particularly among AYP survivors, remains to be seen, given the fear of disclosure from threats from the perpetrators to not being believed, feeling responsible and stigmatisation. Our data support and also add to the current understanding of sexual violence of AYP, providing unique insights to prevention and management of rape and sexual assault in AYP in sub-Saharan Africa, which sadly still needs strategic attention.
